# Structural Characteristic of the Initial Unfolded State on Refolding Determines Catalytic Efficiency of the Folded Protein in Presence of Osmolytes

**DOI:** 10.1371/journal.pone.0109408

**Published:** 2014-10-14

**Authors:** Marina Warepam, Gurumayum Suraj Sharma, Tanveer Ali Dar, Md. Khurshid Alam Khan, Laishram Rajendrakumar Singh

**Affiliations:** 1 Dr. B. R. Ambedkar Center for Biomedical Research, University of Delhi, Delhi, India; 2 Department of Clinical Biochemistry, University of Kashmir, Srinagar, Jammu & Kashmir, India; 3 School of Life Sciences, B.S. Abdur Rahman University, Chennai, India; CNR, Italy

## Abstract

Osmolytes are low molecular weight organic molecules accumulated by organisms to assist proper protein folding, and to provide protection to the structural integrity of proteins under denaturing stress conditions. It is known that osmolyte-induced protein folding is brought by unfavorable interaction of osmolytes with the denatured/unfolded states. The interaction of osmolyte with the native state does not significantly contribute to the osmolyte-induced protein folding. We have therefore investigated if different denatured states of a protein (generated by different denaturing agents) interact differently with the osmolytes to induce protein folding. We observed that osmolyte-assisted refolding of protein obtained from heat-induced denatured state produces native molecules with higher enzyme activity than those initiated from GdmCl- or urea-induced denatured state indicating that the structural property of the initial denatured state during refolding by osmolytes determines the catalytic efficiency of the folded protein molecule. These conclusions have been reached from the systematic measurements of enzymatic kinetic parameters (*K*_m_ and *k*_cat_), thermodynamic stability (*T*_m_ and Δ*H*_m_) and secondary and tertiary structures of the folded native proteins obtained from refolding of various denatured states (due to heat-, urea- and GdmCl-induced denaturation) of RNase-A in the presence of various osmolytes.

## Introduction

Protein folding is remarkably a complex process by which a polymer of amino acids that samples many conformations in its unfolded state adopts a well-packed and essentially a unique native fold. However, many cells and organisms constantly encounter several denaturing hostile stresses that consequently disrupt the fidelity of proper protein folding resulting in the loss of biological functions. Among the major stresses faced by organisms or cells are the variations in water content, perturbed ionic homeostasis, extremes of temperature and osmotic stresses [1], [2], [3]. Nature has developed strategies to ensure that the complex and challenging protein folding process occurs with adequate efficiency and fidelity in the cells. Among the strategies widely employed by a large number of organisms is the accumulation of low molecular weight organic molecules called osmolytes [1], [2], [3]. These osmolytes include polyols (e.g, glycerol, sorbitol), methyl ammonium compounds (e.g, trimethylamine-N-oxide, sarcosine) and free amino acids (e.g, glycine, proline) and derivatives (e.g, taurine, β-alanine). They are found to accumulate up to several millimolar concentrations without altering the macromolecular structure and function [2], [3]. These osmolytes have earlier been shown to (i) increase the protein thermodynamic stability and induce folding of unstructured and intrinsically disordered proteins [4], [5], [6], [7]; (ii) correct misfolding defects of many mutant proteins [8], [9], [10]; (iii) interfere with the formation of protein aggregates [11], [12], [13]. Therefore, osmolytes have been believed to have promising clinical implications against various proteopathies [14], [15], [16].

The molecular mechanism of osmolyte-protein interaction has been widely investigated. It has been known that the preferential hydration effect (or osmophobic force) originating from the unfavorable interaction of osmolyte with the peptide backbone (exposed on protein denaturation) is the main driving force for protein folding [6], [7]. Interaction of osmolyte with the side chains of amino acid residues contributes little (or none) in the osmophobicity. Thus, osmolytes interact primarily with the unfolded state (that has exposed peptide backbones), and not with the native state. In the present study, we have investigated if differences in the structural properties of the denatured states (or unfolded states due to heat-, urea- and GdmCl-induced denaturation) upon refolding in the presence of osmolytes result in the production of different magnitudes of protein folding or catalytic efficiency of the active protein molecules. RNase-A from bovine pancreas was used in this study because of the following reasons: i) it is a classic model protein for refolding studies [17], [18] ii) different denatured states could be generated using different denaturants [19], [20]. We found that protein folding in the presence of osmolytes initiated from the heat-induced denatured state generates native proteins with increased enzyme activity relative to those initiated from the urea- or GdmCl-induced denatured state. The results indicate that the structural property of initial denatured state determines the catalytic efficiency of folded protein molecules.

## Materials and Methods

Commercially lyophilized preparation of Ribonuclease-A (type III-A) from bovine pancreas (RNase-A) was purchased from Sigma Chemical Co. Trimethylamine N-oxide (TMAO), betaine, sarcosine, proline, glycine, β-alanine and cytidine 2′–3′ cyclic monophosphate (C>p) were also purchased from Sigma Chemical Co. Guanidinium chloride (GdmCl) and urea were obtained from MP Biomedicals. These and other chemicals, which were of analytical grade, were used without further purification.

RNase-A solution was dialyzed extensively against 0.1 M KCl solution at pH 7.0 and 4°C. Protein stock solution was filtered using 0.22 µm millipore syringe filter. The protein gave a single band during polyacrylamide gel electrophoresis. Concentration of the protein solution was determined experimentally using molar absorption coefficient, *ε* (M^−1^ cm^−1^) value of 9800 at 277.5 nm [21]. The concentrations of GdmCl and urea stock solutions were determined by refractive index measurements [22]. All solutions for optical measurements were prepared in the degassed 0.05 M cacodylic acid buffer containing 0.1 M KCl. Since pH of the protein solution may change upon addition of the osmolytes, pH of each solution was measured after the denaturation and refolding experiments. It was observed that the change in pH was not significant (∼0.02–0.04).

### Refolding study

Different denatured states of RNase-A were generated by using heat (85°C) and chemical denaturants: GdmCl (6.5 M) and urea (8.5 M) in 0.05 M cacodylic acid buffer at pH 7.0. Protein samples containing the GdmCl (6.5 M) or urea (8.5 M) were incubated overnight at room temperature (25°C). Refolding of the control denatured protein following denaturation experiments were carried out by diluting the denatured protein to a ratio of l: 100 using the same buffer and kept overnight for equilibration. Similarly, refolding in the presence of osmolytes was also carried out by diluting the denatured protein with the buffer that contains desired concentration of the osmolytes. For refolding from heat-induced denaturation, the protein solution in the absence and presence of osmolytes were heated at 85°C for 15 minutes and immediately cool down to 25°C using a dry bath (Indogenix).

To remove osmolytes (especially 1 M of TMAO and sarcosine) from the osmolyte-assisted refolded proteins (obtained from heat-, GdmCl- and urea-induced denatured states), osmolyte containing protein samples were dialyzed for 24 hours against 0.05 M cacodylic acid buffer at pH 7.0 and 4°C.

### Enzyme activity measurements

For determining the effect of osmolytes on the kinetic parameters (*K*_m_, Michaelis constant and *k*_cat_, catalytic constant) activity of native and refolded RNase-A were measured following the procedure described by Crook et al. using cytidine 2′–3′ cyclic monophosphate (C>p) as a substrate [23]. For this, protein (0.035 mg ml^−1^) and substrate in the concentration range 0.05–0.50 mg ml^−1^ were pre-incubated separately in a given osmolyte concentration. RNase-A mediated hydrolysis of the substrate was followed by measuring the change in the absorbance at 292 nm at 25°C for 20 min in Jasco V-660 UV/Vis spectrophotometer and from each progress curve at a given substrate concentration, initial velocity (*v*) was determined from the linear portion (first 30 sec) of the progress curve. The plot of *v* versus [S] (in mM) was analyzed for *K*_m_ and *V*_max_ using equation,
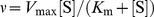
(1)where *v* is the initial velocity, and [S] is the substrate concentration. From this analysis the values of *k*_cat_ were determined.

### Thermal denaturation studies

Thermal denaturation studies of the folded RNase-A obtained from refolding of the heat-, GdmCl- and urea-induced denatured states were carried out using Jasco V-660 UV/Vis spectrophotometer equipped with a Peltier type controller (ETCS-761) with a heating rate of 1°C/min. This scan rate was found to provide adequate time for equilibration. Each sample was heated from 25 to 85°C. Change in the absorbance was followed at 287 nm. After denaturation, the sample was cooled down to measure reversibility. The reversibility was checked by comparing the optical property of the native protein and refolded protein after round of denaturation and was found to be identical (data not shown). All solution blanks showed negligible change in absorbance with temperature and were, therefore, neglected during the data analysis. Each heat-induced transition curve was analyzed for *T*_m_ (the midpoint of heat denaturation) and Δ*H*_m_ (the enthalpy change of denaturation at *T*_m_) using a non linear least-squares analysis equation [24],
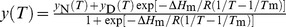
(2)where *y*(*T*) is the optical property at temperature *T* (Kelvin); *y*_N_(*T*) and *y*_D_(*T*) are the optical properties of the native and the denatured protein molecules, respectively; and R is the gas constant. In the analysis of the transition curve, it was assumed that a parabolic function describes the dependence of the optical properties of native and denatured protein molecules i.e., *y*_N_(*T*) = *a*_N_+*b*_N_ *T*+*c*_N_ *T* ^2^, and *y*_D_(*T*) = *a*_D_+*b*_D_ *T+c*_D_ *T* ^2^, where *a*_N_, *b*_N_, *c*_N_, *a*_D_, *b*_D_, and *c*_D_ are temperature-independent coefficients.

### Circular Dichroism measurements

The far- and near-UV circular dichroism (CD) spectra of folded RNase-A obtained from refolding of heat-, GdmCl- and urea-induced denatured states were measured at least three times in a J-810 (Jasco spectropolarimeter) equipped with a Peltier-type temperature controller (Jasco PTC-424S). Each spectrum of the protein was corrected for contribution of its blank. The final concentration of the protein was 0.5 mg/ml. The path length of the cuvette used for far- and near-UV CD measurements were 1.0 mm and 10 mm respectively. The CD signal at each wavelength was converted into mean residue ellipticity (deg cm^2^ dmol^−1^) using the relation,
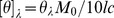
(3)where *θ*_λ_ is the observed ellipticity (millidegrees) at the wavelength λ, *M*_○_ is the mean residue weight of the protein, *c* is the protein concentration (mg/cm^3^), *l* is the path length (centimeters). It should be noted that the CD instrument was routinely calibrated with D-10-camphorsulfonic acid.

### Fluorescence measurements

Fluorescence emission spectra of folded RNase-A obtained from refolding of heat-, GdmCl- and urea-induced denatured states in absence and presence of 1 M of each osmolyte was measured at least three times in a Perkin Elmer-LS 55 (Fluorescence spectrometer). The final concentration of the protein was 1.5 µM. The path length of the cuvette used for fluorescence measurements was 5.0 mm. The excitation wavelength was 268 nm and the emission spectra were recorded from 290–400 nm. All necessary background corrections were made.

## Results

**Figure 1:**
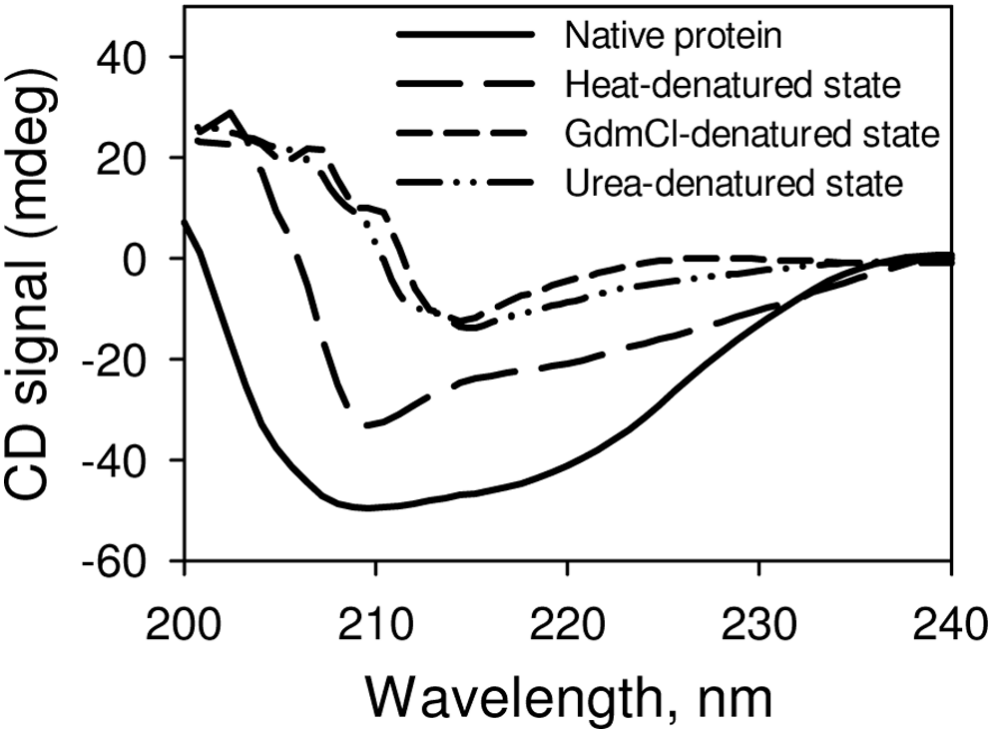
Secondary structural characteristic of various denatured states of RNase-A at pH 7.0. The GdmCl- and urea- induced denatured states were generated using 6.5 M GdmCl and 8.5 M urea respectively. The heat induced-denatured state was generated by incubating the protein at 85°C for 15 minutes.

**Figure 2:**
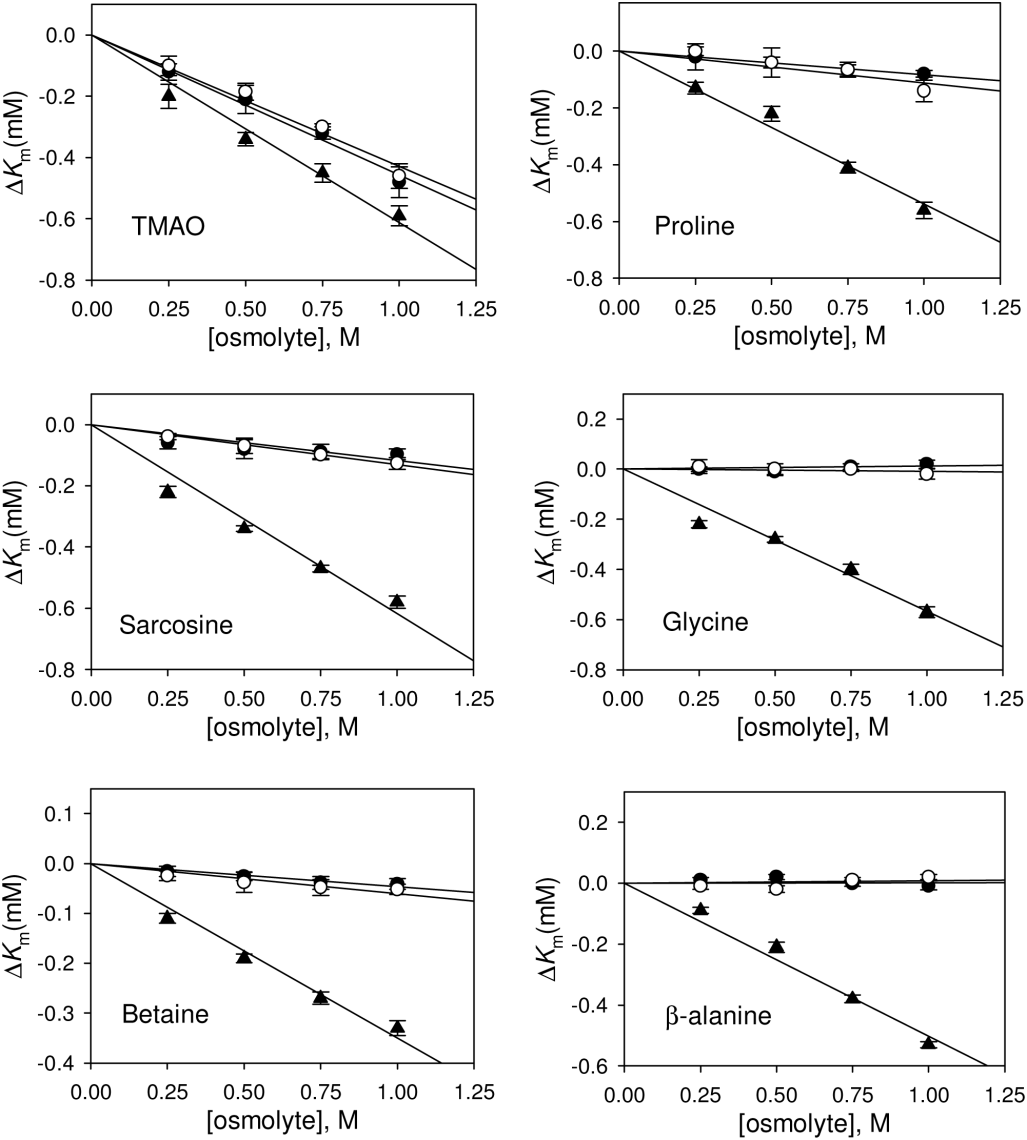
Effect of osmolytes on *K*_m_ of RNase-A. Plot of Δ*K*_m_ versus [osmolyte]. Δ*K*_m_ of folded RNase-A obtained from refolding of heat-, GdmCl- and urea-induced denatured states are represented by (▴), (•) and (○), respectively.

**Figure 3:**
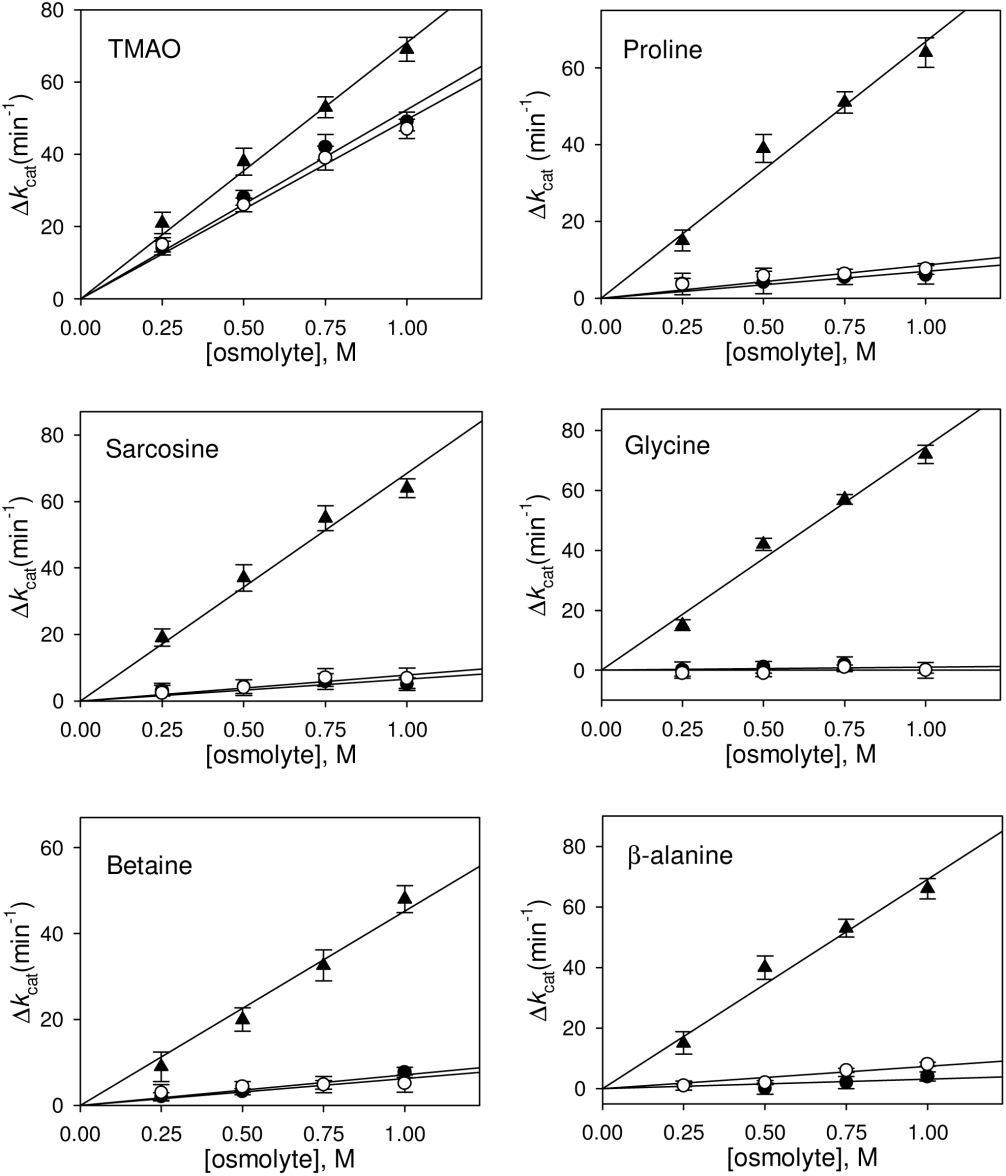
Effect of osmolytes on *k*_cat_ of RNase-A. Plot of Δ*k*_cat_ versus [osmolyte]. Δ*k*_cat_ of folded RNase-A obtained from refolding of heat-, GdmCl- and urea-induced denatured states are represented by (▴), (•) and (○), respectively.

Heat, GdmCl or urea has earlier been known to induce different denatured states having different structural properties. Heat-induced denatured state retains a large amount of residual secondary structures while GdmCl or urea induces a random coil denatured conformation [25], [26], [27]. To investigate the effect of different osmolytes (proline, glycine, β-alanine, TMAO, sarcosine, betaine) on protein folding, we have chosen RNase-A and generated its different denatured states using different denaturing agents (heat, GdmCl, urea) (see Figure 1). It is clearly seen in this figure that heat-induced denatured state is different from that of either GdmCl- or urea-induced denatured state. Table 1 shows the measured enzymatic kinetic parameters (*K*_m_ and *k*_cat_) of the folded proteins obtained from refolding of the respective heat-, GdmCl- or urea-induced denatured state. We have compared the change in *K*_m_ and *k*_cat_ of refolded proteins obtained from the three different denatured states in the presence of osmolytes by plotting Δ*K*_m_ versus osmolyte concentration (see Figure 2)and Δ*k*_cat_ versus osmolyte concentration (see Figure 3). It is seen in these figures that the activity of the folded protein obtained from osmolyte-assisted refolding of heat-induced denatured state has a decreased *K*_m_ and an increased *k*_cat_ of the RNase-A-mediated hydrolysis of cytidine 2′–3′ cyclic mono phosphate (c>p). On the other hand, *K*_m_ and *k*_cat_ values of the folded proteins obtained from the osmolyte-assisted refolding of GdmCl- or urea-induced denatured state remain unchanged (except in case of TMAO). Furthermore, Table 2 shows the measured *K*_m_ and *k*_cat_ values of the native RNase-A in presence of 1 M osmolytes.

**Table 1:** Activity parameters of folded RNase-A obtained from refolding of the Heat-, GdmCl- and Urea-denatured states in the presence of osmolytes at pH 7.0 and 25°C^a^^,^ b.

[osmolyte]		TMAO		Sarcosine		Betaine		Proline		Glycine		β-alanine	
	M	*K*_m_	*k*_cat_	*K*_m_	*k*_cat_	*K*_m_	*k*_cat_	*K*_m_	*k*_cat_	*K*_m_	*k*_cat_	*K*_m_	*k*_cat_
**Native Control**	0.00	1.31	192	1.31	192	1.31	192	1.31	192	1.31	192	1.31	192
**Refolded from Heat-denatured state**													
	0.00	1.30	190	1.30	190	1.30	190	1.30	190	1.30	190	1.30	190
	0.25	1.07	212	1.11	210	1.18	200	1.16	206	1.07	201	1.20	203
	0.50	0.91	230	1.05	231	1.10	211	1.07	238	1.01	233	1.11	231
	0.75	0.84	244	0.90	249	1.02	219	0.88	247	0.89	248	0.91	244
	1.00	0.71	260	0.74	255	0.96	239	0.73	259	0.72	263	0.76	261
Values obtained after dialysis*		0.70	263	0.72	257								
**Refolded from GdmCl-denatured state**													
	0.00	1.29	191	1.29	191	1.29	191	1.29	191	1.29	191	1.29	191
	0.25	1.17	208	1.28	194	1.31	191	1.29	193	1.29	191	1.30	192
	0.50	1.12	220	1.27	196	1.29	192	1.27	195	1.28	192	1.31	191
	0.75	0.97	233	1.27	195	1.27	195	1.26	196	1.30	193	1.29	193
	1.00	0.81	240	1.26	197	1.25	196	1.24	198	1.31	192	1.28	195
Values obtained after dialysis*		0.77	239	1.22	199								
**Refolded from Urea-denatured state**													
	0.00	1.30	191	1.30	191	1.30	191	1.30	191	1.30	191	1.30	191
	0.25	1.19	208	1.30	195	1.32	192	1.29	193	1.30	190	1.28	192
	0.50	1.15	219	1.31	196	1.31	193	1.27	194	1.29	190	1.27	193
	0.75	0.99	230	1.28	198	1.28	193	1.26	198	1.29	191	1.30	197
	1.00	0.83	238	1.26	198	1.26	194	1.23	199	1.27	192	1.31	199
Values obtained after dialysis*		0.84	233	1.26	195								

a Errors in *K*_m_ and *k*_cat_ from triplicate measurements are 8–10%.

b The Units of *K*_m_ and *k*_cat_ are mM and min^−1^, respectively.

*These values were obtained from enzymatic measurements after extensive dialysis for 24 hours.

**Table 2:** Activity parameters of native RNase-A (without refolding) in presence of 1 M osmolytes at pH 7.0 and 25°C.

	*K*_m_ (mM)	*k*_cat_ (min^−1^)
Native control (0 M)	1.31±0.11	192±18.6
TMAO	0.57±0.09	246±22.1
Sarcosine	0.61±0.07	248±19.8
Betaine	0.80±0.08	215±15.3
Proline	1.19±0.10	194±17.2
Glycine	1.14±0.13	189±18.1
β-alanine	1.16±0.10	190±17.9

**Figure 4:**
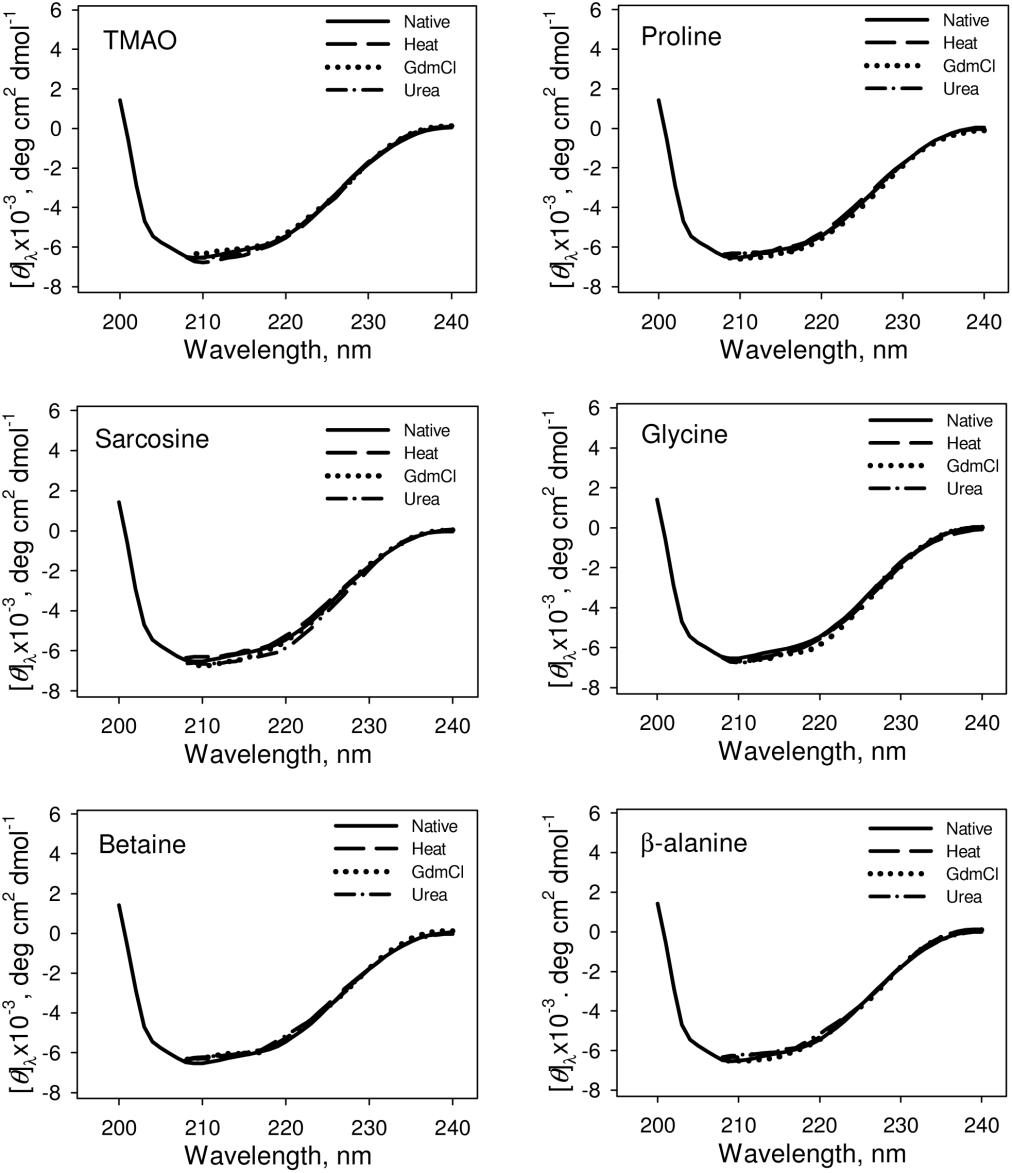
Secondary structural characteristic of the folded RNase-A at pH 7.0 and 25°C. Representative CD spectra of folded RNase-A (from heat-, GdmCl-, urea-induced denatured states) obtained from refolding in the presence of 1 M osmolytes. The CD spectra of the refolded RNase-A in the absence of osmolytes is identical with the native CD spectra and is omitted. Therefore, we have shown spectra only for the native (without refolding) control in this figure.

**Figure 5:**
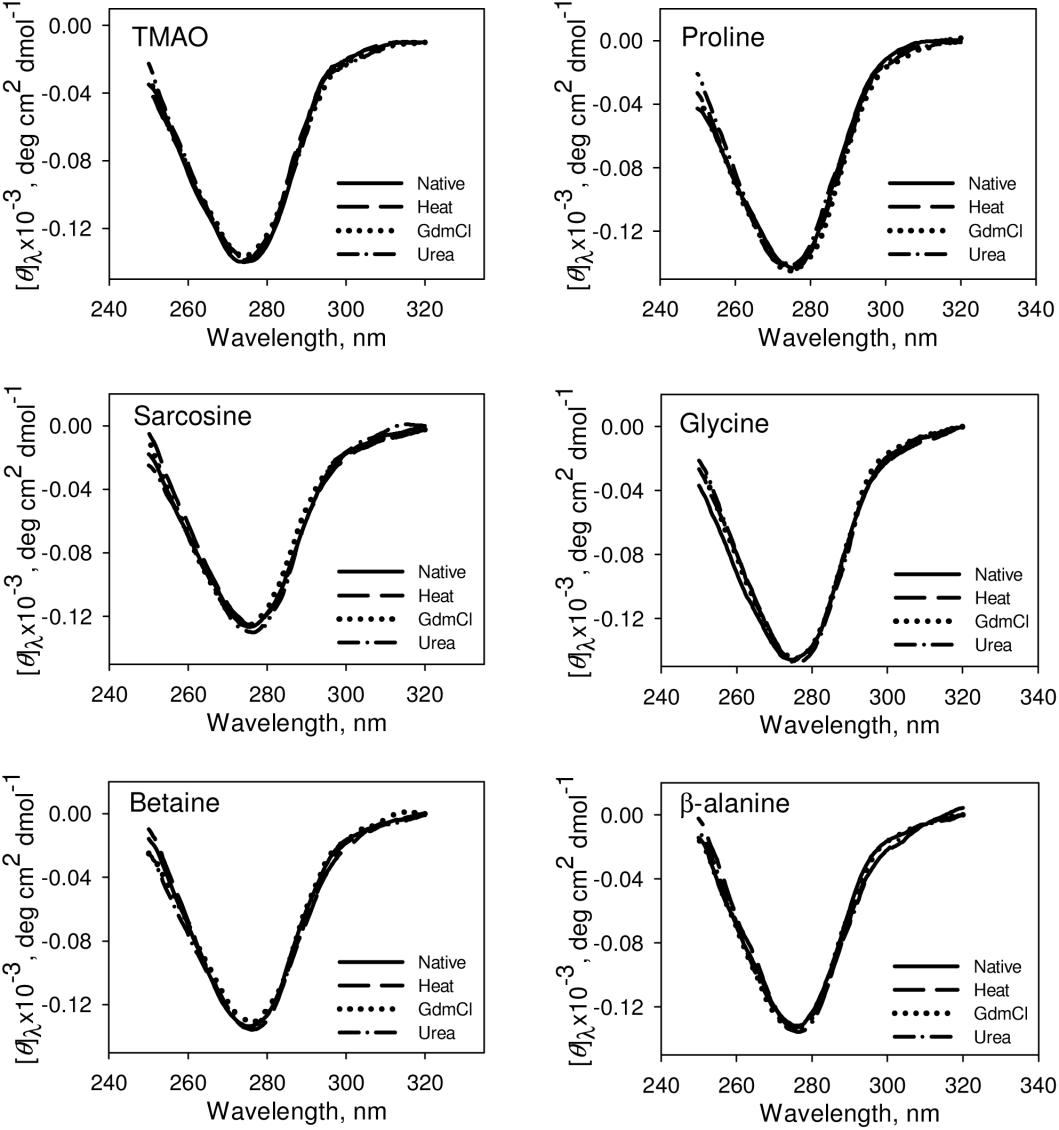
Tertiary structural characteristic of folded RNase-A at pH 7.0 and 25°C. Representative CD spectra of folded RNase-A (from heat-, GdmCl-, urea-induced denatured states) obtained from refolding in the presence of 1 M osmolytes. The CD spectra of the refolded RNase-A in the absence of osmolytes is identical with the native CD spectra and is omitted. Therefore, we have shown spectra only for the native (without refolding) control in this figure.

**Figure 6:**
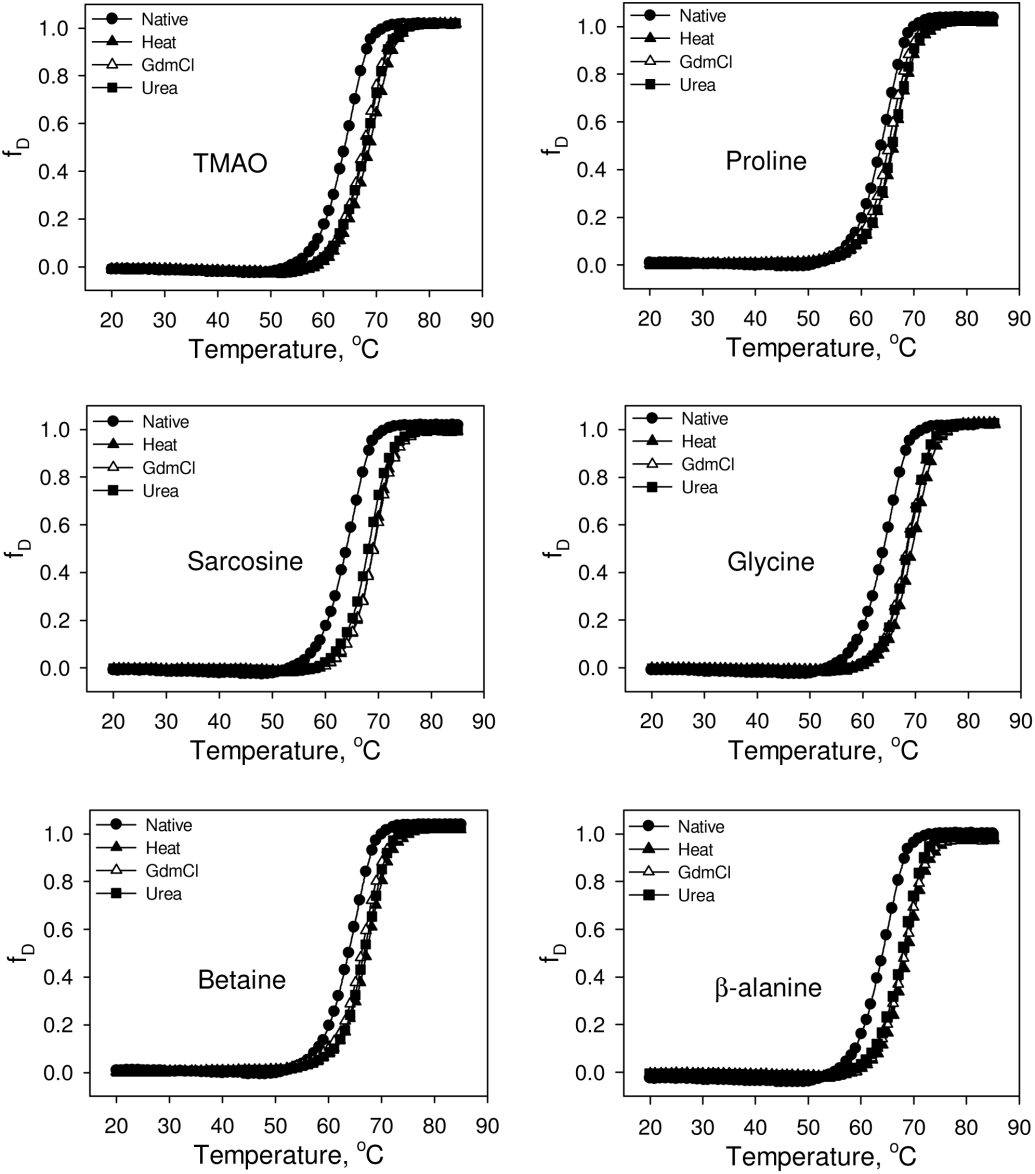
Effect of osmolytes on the stability of folded RNase-A at pH 7.0. Representative thermal denaturation curves of folded RNase-A obtained from refolding of various denatured states in the presence of 1 M osmolytes. Denaturation curves of the refolded RNase-A (from heat-, GdmCl-, urea-induced denatured states) in the absence of osmolytes are identical with the native (without refolding) transition curve. Therefore, we have shown only the transition curve of the native (without refolding) protein.

We have further investigated the native state structure of the folded proteins obtained from osmolyte-assisted refolding of different denatured states. Figure 4 and Figure 5 respectively show the effect of osmolytes on the secondary and tertiary structures of the refolded proteins. It is seen in these figures that both the secondary and tertiary structure of the osmolyte-induced folded native proteins obtained from the three different denatured states are identical. It is also seen in Figure S1 that there is no significant alteration in the environment of tyrosine of folded RNase-A obtained from the three different denatured states in presence of osmolytes. Figure 6 shows the heat-induced denaturation profiles of the folded proteins obtained from the osmolyte-assisted refolding of the different denatured states. The evaluated thermodynamic parameters (*T*_m_ and Δ*H*_m_) are presented in Table 3. It is seen in Figure 6 and Table 3 that at a given osmolyte concentration there is no significant difference in the thermodynamic stability (in terms of *T*_m_ and Δ*H*_m_) of the folded RNase-A obtained from osmolyte-assisted refolding of the three different denatured states. The results indicate that the observed increase in activity of the folded protein (in terms of decrease in *K*_m_ and increase in *k*_cat_) obtained from the osmolyte-assisted refolding of the heat-induced denatured state relative to those obtained from refolding of the GdmCl- or urea-induced denatured state cannot be explained in terms of structure and stability of the characteristics of the folded protein.

**Table 3:** Thermodynamic parameters of folded RNase-A obtained from refolding of the Heat-, GdmCl- and Urea-denatured states in the presence of osmolytes at pH 7.0^a^^,^ b.

		TMAO		Sarcosine		Betaine		Proline		Glycine		β-alanine	
[osmolyte]		*T*_m_	Δ*H*_m_	*T*_m_	Δ*H*_m_	*T*_m_	Δ*H*_m_	*T*_m_	Δ*H*_m_	*T*_m_	Δ*H*_m_	*T*_m_	Δ*H*_m_
**Native Control**	0.00 M	64.1	111	64.1	111	64.1	111	64.1	111	64.1	111	64.1	111
**Refolded from Heat-denatured state**													
	0.00 M	63.7	110	63.7	110	63.7	110	63.7	110	63.7	110	63.7	110
	0.25 M	64.3	117	64.9	119	64.1	113	64.2	116	66.8	120	63.8	116
	0.50 M	65.1	119	65.5	121	64.9	114	64.9	117	67.2	120	64.3	117
	0.75 M	66.2	121	66.8	125	66.2	116	65.3	117	68.0	121	66.0	118
	1.00 M	68.1	123	68.5	129	67.1	119	66.2	120	69.1	122	68.2	119
**Refolded from GdmCl-denatured state**													
	0.00 M	63.9	111	63.9	111	63.9	111	63.9	111	63.9	111	63.9	111
	0.25 M	64.2	116	64.3	117	63.8	114	63.1	117	66.0	118	64.0	117
	0.50 M	64.9	119	65.8	122	64.7	115	63.5	117	66.9	119	64.6	117
	0.75 M	66.0	120	66.5	124	65.6	116	64.9	120	67.4	120	65.2	118
	1.00 M	67.5	121	68.3	127	66.5	118	65.4	121	68.2	122	67.7	119
**Refolded from Urea-denatured state**													
	0.00 M	63.5	110	63.5	110	63.5	110	63.5	110	63.5	110	63.5	110
	0.25 M	63.7	117	64.2	118	64.0	115	64.2	116	65.1	119	63.7	116
	0.50 M	64.1	118	65.2	120	64.6	115	64.9	117	65.7	118	64.4	117
	0.75 M	65.6	120	66.7	124	65.8	117	65.1	118	66.8	121	65.0	118
	1.00 M	67.2	122	67.8	127	66.7	117	65.6	119	68.3	122	67.5	119

a Errors in *T*_m_ and Δ*H*_m_ from triplicate measurements are 0.1–0.6% and 2–5%, respectively.

b Units of *T*_m_ and Δ*H*_m_ are °C and kcal mol^−1^, respectively.

## Discussion

It has been well known that osmolyte induces protein folding due to the unfavorable interaction of osmolyte with the protein peptide backbone. Such osmolyte-induced protein folding is brought about by the effect of osmolyte on the unfolded state (denatured state) not the folded, native state of the protein as the protein peptide backbones are not exposed to the surface in case of native protein indicating crucial importance of the denatured states in osmolyte-assisted protein folding process. We have therefore, investigated if different denatured states of a protein (induced by different denaturing agents) interact differently with the osmolytes to induce protein folding. For this, we have intentionally denatured RNase-A using different denaturing agents (urea, GdmCl and heat). As revealed by far-UV CD measurements, we observed no significant differences in the structural characteristic of the denatured states induced by 6.5 M GdmCl and 8.5 M urea. However, the heat-induced denatured state (at 85°C) has been found to retain significant amount of secondary structures (see Figure 1). In agreement to this observation heat-induced denatured state of many proteins (including RNase-A) has been reported to have different structure, heat capacity change and solvent accessibility different from that of either GdmCl- or urea-induced denatured state [20], [26], [27], [28], [29]. We have further analyzed for the change in the secondary structures (α-helix and β-sheet) due to each type of denaturation following the method described by Yang et al. [30]. The percent α-helix and β-sheet retained in heat-induced denatured state were 12% and 16% respectively while the percent changes in the type of the secondary structures for the GdmCl- and urea-induced denatured states were almost identical (3–3.5% for α-helix and 5–6% β-sheet). In addition, β-sheet was found to be little more sensitive than α-helix in each type of denatured state (see Table S1). We have further investigated if refolding initiated from the different denatured states of RNase-A (induced by GdmCl, urea and heat) in the presence of osmolytes results in the production of folded native states that have different catalytic efficiencies by measuring functional activity parameters (*K*_m_ & *k*_cat_) of the enzyme (see Table 1). The agreement between the *K*_m_ and *k*_cat_ values of the folded native protein obtained from the refolding studies (refolded protein in the absence of osmolytes) with that of the native, folded protein (without refolding) suggests that our measurements of enzymatic kinetics parameters are authentic and accurate [31]. It is also seen in Table 1, Figure 2 and 3 that values of *K*_m_ and *k*_cat_ of the folded protein obtained from osmolyte-assisted refolding of the GdmCl- or urea-denatured state are almost identical with that of the control (refolded protein in the absence of osmolytes) except for TMAO. An increase in the enzyme activity of refolded RNase-A in presence of TMAO indicates that the folded native state induced by TMAO is catalytically different from those induced by other osmolytes. Perhaps TMAO might have induced catalytic sites in folded RNase-A that are more appropriate for substrate binding than those induced by other osmolytes. In support of this argument, it has been shown that in presence of TMAO (as compared to that of glycerol) the catalytic sites of trypsin are folded in such a manner that they are more suited for substrate binding and hence enhanced enzyme activity [32]. In contrast to other osmolytes, TMAO has also earned special attention for its unique capability to induce folding of denatured state to folded native state that has significant functional activity [33], [34]. Furthermore, we observed that the activity of folded protein obtained from osmolyte-assisted refolding of the heat-induced denatured state results in increased enzyme activity (due to decrease in *K*_m_ and increase in *k*_cat_) relative to those obtained from the osmolyte-assisted refolding of the GdmCl- or urea-induced denatured state. It may be noted that the presence of osmolytes in the refolded protein solutions might influence the observed kinetic parameters. To investigate for this, we have further measured activity parameters (*K*_m_ and *k*_cat_) of the osmolyte-assisted refolded proteins from the three different denatured states (for TMAO and sarcosine only) after removal of the osmolytes by extensive dialysis. It was observed that there is no significant difference between the values of *K*_m_ and *k*_cat_ of the refolded proteins before and after the dialysis (see Table 1) suggesting that the presence of osmolytes does not influence the catalytic behavior of the refolded proteins. It is also important for us to compare the differences in the enzymatic parameters of the refolded proteins (from different denatured states in the presence of osmolytes) with that of the native protein in the presence of osmolytes. For this, we have intentionally measured the *K*_m_ and *k*_cat_ of the native RNase-A (without refolding) in the presence of 1 M of each of the osmolyte. We found that there exist differences in the activity parameters of the refolded protein obtained from refolding of heat-induced denatured state and that of the native protein in the presence of 1 M of the respective osmolytes (see Table 2). The results indicate that osmolyte-induced refolded proteins obtained from heat-induced denatured state generate active enzyme molecules that is different from those of other osmolyte-induced refolded proteins (from GdmCl- or urea-induced denatured state) and native proteins (in the presence of osmolytes). Thus, it is known from the activity measurements that folded protein obtained from osmolyte-assisted refolding of the heat-induced denatured state generates catalytically more active protein molecules as compared to that obtained from the refolding of the GdmCl- or urea-denatured state.

We have further investigated for the possible reason behind the differences in the activity of the folded proteins obtained from osmolyte-assisted refolding from various denatured states. One possibility is that the differences in the activity of the folded proteins obtained from osmolyte-assisted refolding of different denatured states is due to their different extents of folding leading to the formation of different native states. To investigate for this possibility, we have measured the secondary and tertiary structures of the folded proteins obtained from osmolyte-assisted refolding from different denatured states. It is seen in Figure 4, 5 and S1 that both the secondary and tertiary structures of the folded proteins are similar to that of the control (spectra of native protein in the absence of osmolytes), indicating that the folded protein molecules do not differ in the structural properties, and hence might be of similar native state. Another possibility is that the thermodynamic stability of folded protein obtained from osmolyte-assisted refolding of the heat-induced denatured state might be higher than that obtained from refolding of GdmCl- or urea-induced denatured state. Our results given in Table 3 and Figure 6 suggest that at a given osmolyte concentration thermodynamic stabilities of the folded proteins obtained from osmolyte-assisted refolding of heat-, GdmCl- and urea-induced denatured states (in terms of *T*_m_ and Δ*H*_m_) are almost identical (although different from the refolded control in absence of osmolytes). The results indicate that there is no difference in the number of active enzyme molecules generated due to osmolyte-assisted refolding. Taken together, both the structural and thermodynamic data could not explain for the observed differences in the activity of RNase-A obtained from the osmolyte-assisted refolding from the three different denatured states. However, we do not rule out the fact that at different osmolyte concentrations *T*_m_ of the folded protein is changed which might be due to some small effect on the denaturation equilibrium, N conformation ↔ D conformation, towards the left [35], [36].

Lastly, one best possibility is that the osmolyte-assisted refolding processes might have affected the properties of the microstates of different refolded proteins. Interestingly, it has been reported that osmolytes can affect the microstates of protein resulting in the altered enzymatic activity [37], [38], [39]. Indeed different microstates (having low and high activity state) exist in a native state of a protein [37], [38], [39], [40]. Therefore, we speculate that osmolyte-assisted refolding can go at least in two different folding pathways. One that initiates from the structured denatured state might have produced folded protein molecules in the higher activity state. While the other refolding initiated from the unstructured denatured state results in the production of folded protein molecules with low activity state. In support of the first argument, it has been shown that presence of residual structure in the denatured state predisposes the protein to fold efficiently to its native structure [41]. Furthermore, residual structures ideally can limit the conformational search towards a thermodynamic (or kinetic) minimum to increase protein folding efficiency [42]. Thus, our results indicate that the differences in the structural characteristic of the initial denatured states during osmolyte-assisted protein folding determine the catalytic efficiency of the folded native proteins. Intracellularly, it might be possible that different stress conditions induce different denatured states of a protein leading to protein folding in different pathways due to osmolytes that indeed might help to regulate the functional activity of enzymes under stressful conditions.

## Supporting Information

Figure S1 **Tyrosine fluorescence spectra of folded RNase-A at pH 7.0 and 25°C.** Fluorescence emission spectra of folded RNase-A (from heat-, GdmCl-, urea-induced denatured states) obtained from refolding in the presence of 1 M of each osmolyte. The fluorescence emission spectra of the refolded RNase-A in the absence of osmolytes is identical with the native fluorescence emission spectra and is omitted. Therefore, we have shown spectra only for the native (without refolding) control in this figure.
(TIF)

Table S1 **Comparison of secondary structural content of the different denatured states of RNase-A.**
(DOCX)
