# Supplementary figures and images for: Structural Characteristic of the Initial Unfolded State on Refolding Determines Catalytic Efficiency of the Folded Protein in Presence of Osmolytes
Source: PLoS One. 2014 Oct 14;9(10):e109408. doi: 10.1371/journal.pone.0109408 (PMC4196897; doi:10.1371/journal.pone.0109408)

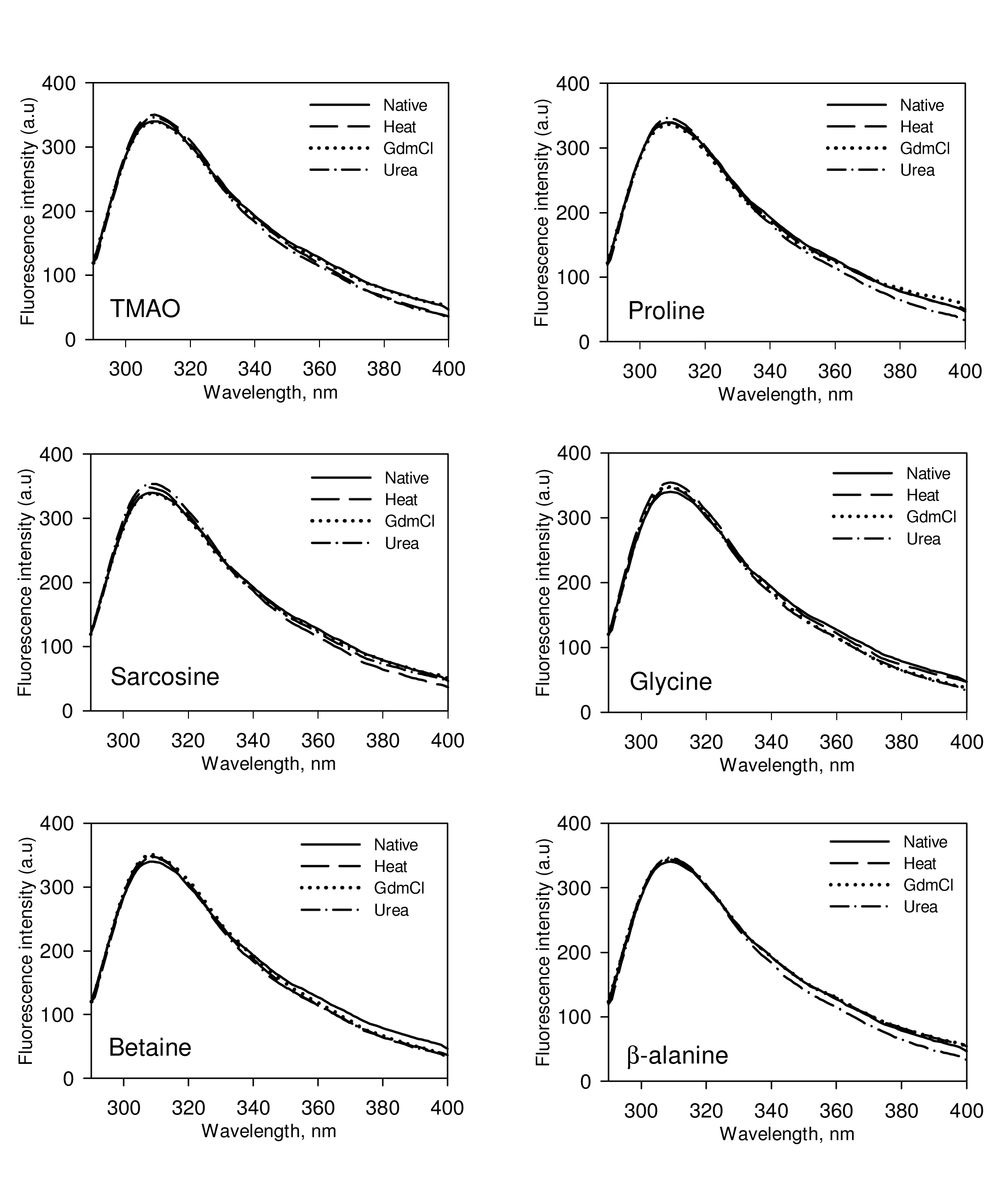

Supplement: Figure S1 — Tyrosine fluorescence spectra of folded RNase-A at pH 7.0 and 25°C. Fluorescence emission spectra of folded RNase-A (from heat-, GdmCl-, urea-induced denatured states) obtained from refolding in the presence of 1 M of each osmolyte. The fluorescence emission spectra of the refolded RNase-A in the absence of osmolytes is identical with the native fluorescence emission spectra and is omitted. Therefore, we have shown spectra only for the native (without refolding) control in this figure. (TIF) [file pone.0109408.s001.tif]
