# Supplementary material for: Structural Characteristic of the Initial Unfolded State on Refolding Determines Catalytic Efficiency of the Folded Protein in Presence of Osmolytes
Source: PLoS One. 2014 Oct 14;9(10):e109408. doi: 10.1371/journal.pone.0109408 (PMC4196897; doi:10.1371/journal.pone.0109408)
Supplement: Table S1 — Comparison of secondary structural content of the different denatured states of RNase-A. (DOCX) [file pone.0109408.s002.docx]

|  | **% α-helix** | **% β-sheet** |
| --- | --- | --- |
| Native state | 19.4 | 38 |
| Heat denatured state | 12 | 16 |
| Gdmcl denatured state | 3.0 | 5.1 |
| Urea denatured state | 3.4 | 5.8 |
